# A machine learning model based on preoperative multiparametric quantitative DWI can effectively predict the survival and recurrence risk of pancreatic ductal adenocarcinoma

**DOI:** 10.1186/s13244-025-01915-9

**Published:** 2025-02-17

**Authors:** Chao Qu, Piaoe Zeng, Changlei Li, Weiyu Hu, Dongxia Yang, Hangyan Wang, Huishu Yuan, Jingyu Cao, Dianrong Xiu

**Affiliations:** 1 Department of Hepatobiliary and Pancreatic Surgery, The Affiliated Hospital of Qingdao University, Qingdao, China; 2 Department of General Surgery, Peking University Third Hospital, Beijing, China; 3 Department of Radiology, Peking University Third Hospital, Beijing, China

**Keywords:** Pancreatic ductal adenocarcinoma, Diffusion-weighted imaging, Machine learning, Prognosis, Prediction model

## Abstract

**Purpose:**

To develop a machine learning (ML) model combining preoperative multiparametric diffusion-weighted imaging (DWI) and clinical features to better predict overall survival (OS) and recurrence-free survival (RFS) following radical surgery for pancreatic ductal adenocarcinoma (PDAC).

**Materials and methods:**

A retrospective analysis was conducted on 234 PDAC patients who underwent radical resection at two centers. Among 101 ML models tested for predicting postoperative OS and RFS, the best-performing model was identified based on comprehensive evaluation metrics, including *C*-index, Brier scores, AUC curves, clinical decision curves, and calibration curves. This model’s risk stratification capability was further validated using Kaplan–Meier survival analysis.

**Results:**

The random survival forest model achieved the highest *C*-index (0.828/0.723 for OS and 0.781/0.747 for RFS in training/validation cohorts). Incorporating nine key factors—*D* value, T-stage, ADC-value, postoperative 7th day CA19-9 level, AJCC stage, tumor differentiation, type of operation, tumor location, and age—optimized the model’s predictive accuracy. The model had integrated Brier score below 0.13 and C/D AUC values above 0.85 for both OS and RFS predictions. It also outperformed traditional models in predictive ability and clinical benefit, as shown by clinical decision curves. Calibration curves confirmed good predictive consistency. Using cut-off scores of 16.73/29.05 for OS/RFS, Kaplan–Meier analysis revealed significant prognostic differences between risk groups (*p* < 0.0001), highlighting the model’s robust risk prediction and stratification capabilities.

**Conclusion:**

The random survival forest model, combining DWI and clinical features, accurately predicts survival and recurrence risk after radical resection of PDAC and effectively stratifies risk to guide clinical treatment.

**Critical relevance statement:**

The construction of 101 ML models based on multiparametric quantitative DWI combined with clinical variables has enhanced the prediction performance for survival and recurrence risks in patients undergoing radical resection for PDAC.

**Key Points:**

- This study first develops DWI-based radiological–clinical ML models predicting PDAC prognosis.
- Among 101 models, RFS is the best and outperforms other traditional models.
- Multiparametric DWI is the key prognostic predictor, with model interpretations through SurvSHAP.

**Graphical Abstract:**

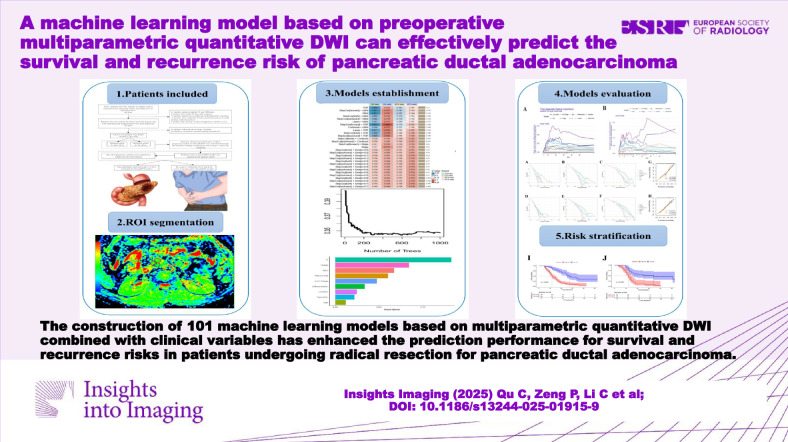

## Introduction

Pancreatic ductal adenocarcinoma (PDAC) is a highly aggressive malignancy, ranking fourth in cancer-related mortality worldwide [1]. Due to early and subtle invasion of surrounding tissues, only about 10–25% of patients have the opportunity to undergo radical resection [2]. Even so, most patients experience postoperative recurrence, leading to a dismal 5-year survival rate of 10–25% [3]. Thus, there is an urgent need for timely and effective risk stratification to enable individualized and precise treatment. However, current research predominantly focuses on the predictive value of clinicopathological data, often yielding suboptimal results due to high tumor heterogeneity [4]. The potential predictive value of preoperative imaging data, particularly from diffusion-weighted imaging (DWI), remains underexplored.

Previous research at our center has suggested that quantitative parameters from diffusion-weighted magnetic resonance imaging (DWI) may play an important role in predicting survival [5]. DWI is non-invasive and its mono-exponential model quantitative parameter, apparent diffusion coefficient (ADC) has been used for the diagnosis of various tumors [6], prediction of malignancy, and prognosis [7]. The development of DWI, not only includes mono-exponential models, but also non-Gaussian fitting models, namely bi-exponential intravoxel incoherent motion and stretched-exponential model. They can provide multiple quantitative parameters for evaluation, such as pure molecular diffusion coefficient (*D* value) and perfusion fraction (*f* value) [8]. Currently, the value of multiparametric quantitative DWI in guiding disease diagnosis and treatment is gradually being recognized, but most studies are still in the preliminary exploration stage [9]. To date, there are no established models using multiparametric quantitative DWI to predict survival and recurrence risk after radical resection of PDAC.

Therefore, building on our previous research, this study aims to develop 101 prognostic models based on machine learning (ML) algorithms using clinical and DWI data from two centers. The optimal radiological–clinical predictive model will be selected through cohort validation and model performance evaluation. The findings will enhance risk stratification for patients undergoing radical resection of PDAC and support the implementation of individualized precision treatment.

## Materials and methods

### Patients

This study retrospectively collected clinical and DWI data from 336 patients diagnosed with PDAC at two pancreatic centers between May 2012 and December 2019, all of whom were scheduled for radical resection surgery.

The inclusion criteria included: (1) complete enhanced CT and MRI data, including multiparametric quantitative DWI, within 14 days before surgery; (2) solid pancreatic tumor diameter greater than 1.5 cm to ensure accurate MRI quantitative parameter measurement; (3) the MRI was of good quality, without obvious artifacts and not seriously affected by other intra-abdominal organs; (4) patients with no other severe heart, liver, lung, or kidney disease.

The exclusion criteria included: (1) neoadjuvant treatment before surgery; (2) preoperative assessment of distant metastasis or inability to undergo radical surgical resection; (3) history of other malignant tumors before surgery; (4) death within 90 postoperative days or incomplete follow-up data.

**Fig. 1:**
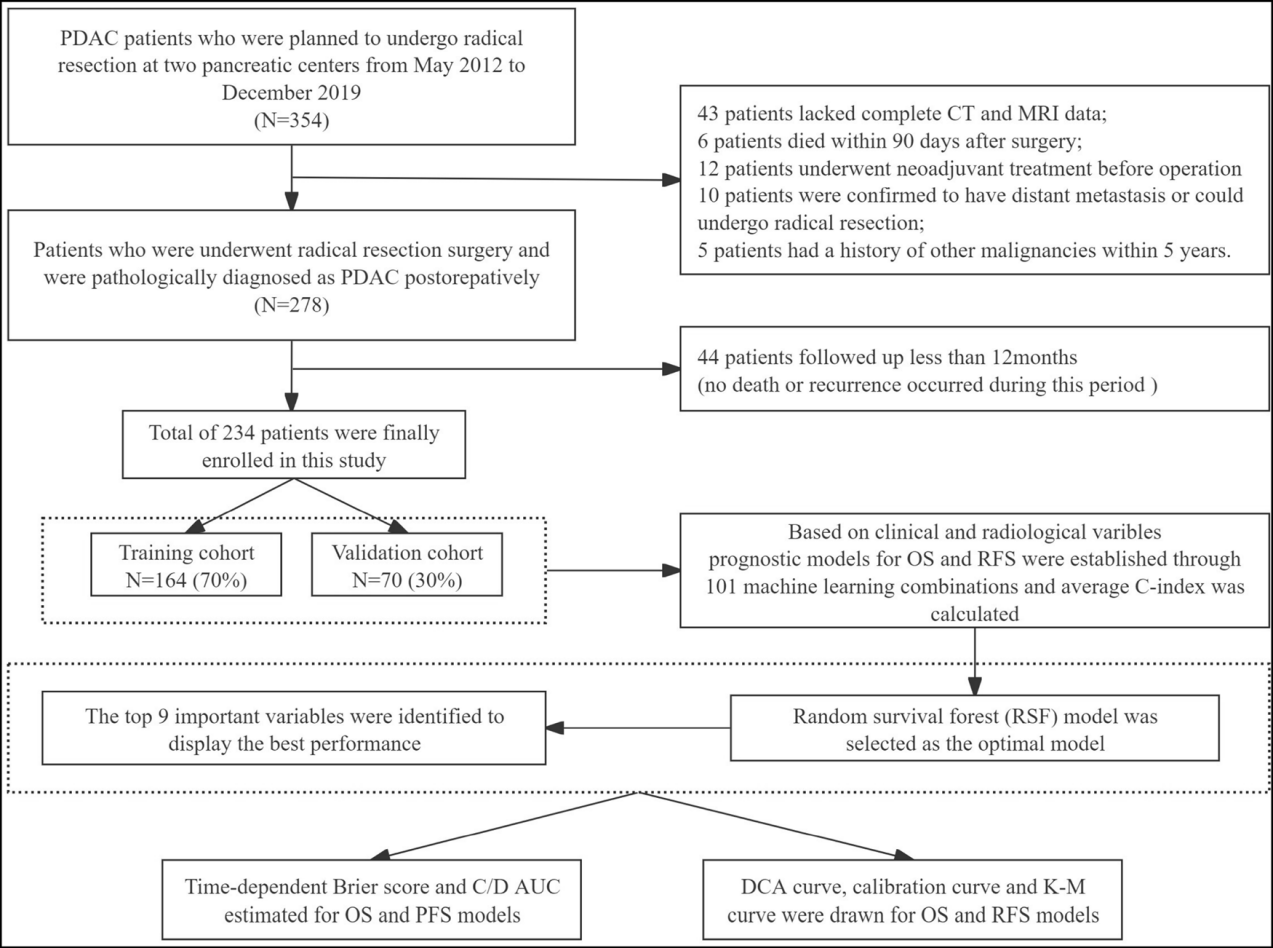
Flowchart for patient inclusion and model establishment

Based on these criteria, 234 patients were included in the study. These patients were then randomly allocated into two groups: the training cohort (*n* = 164) and the validation cohort (*n* = 70), maintaining a 7:3 ratio (Fig. 1). The sample size was sufficient to meet the needs of this research, with the estimation results detailed in Fig. S1. The study was approved by our institutional ethics review board (NO. LM2021120).

### Magnetic resonance imaging analysis

MRI scans for both study cohorts were acquired using a GE Discovery MR750 3.0-T MRI scanner with an 8-channel abdominal coil. The DWI data were postprocessed with built-in software (Functool MADC, GE), applying mono-exponential, bi-exponential, and stretched-exponential models to calculate ADC, *D*, D^*^, *f*, DDC, and α maps (detailed MRI parameter settings and descriptions are available in the supplementary information).

**Fig. 2:**
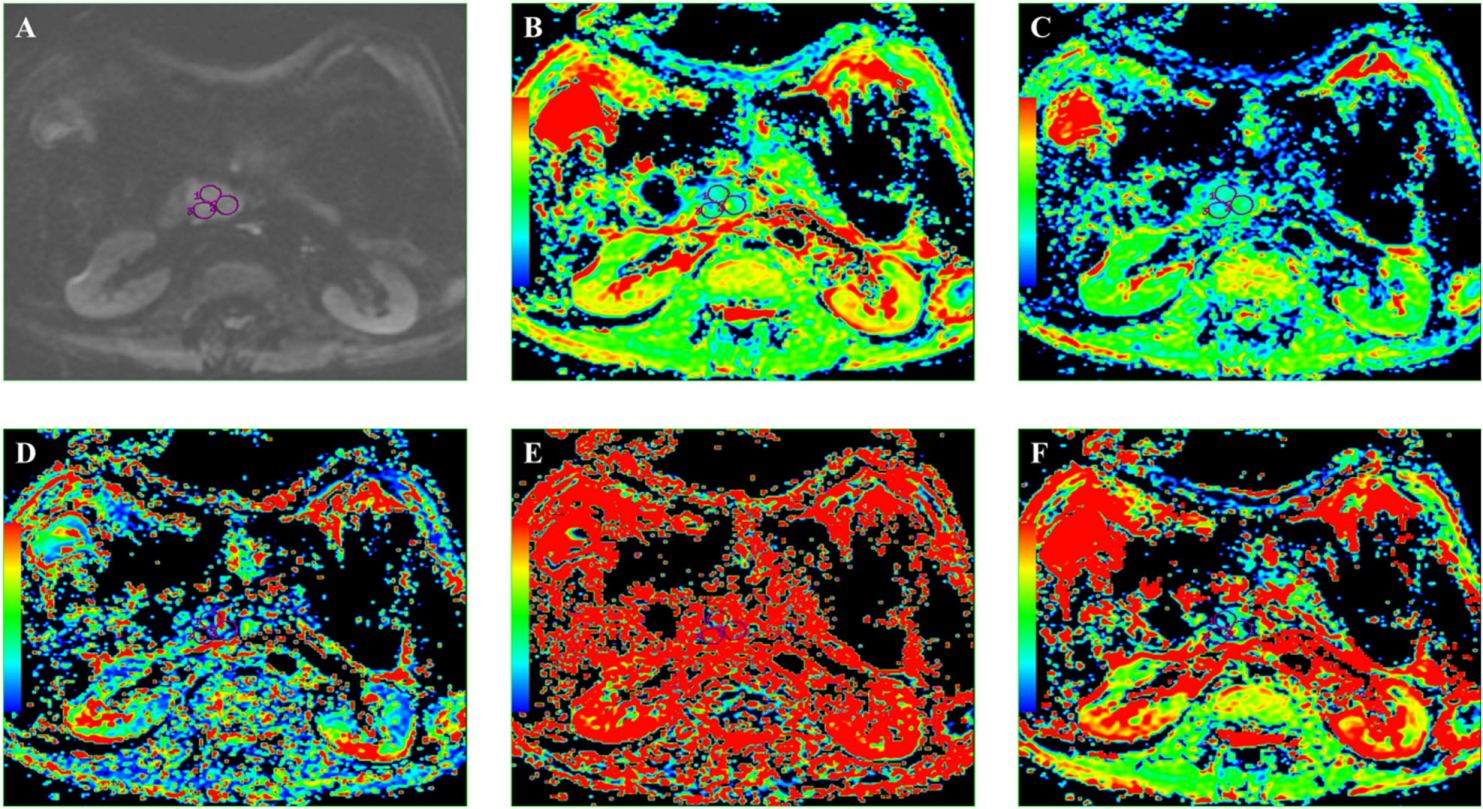
DWI image (**A**), corresponding ADC map (**B**), *D* map (**C**), D^*^ map (**D**), *f* map (**E**), and DDC map (**F**) with corresponding ROI measurements

DWI-derived parameters were analyzed by two radiologists with 12 years and 5 years of post-training experience, respectively, who were blinded to histopathological data. The data were disjointed at the level of patients, images, series, or images. Interobserver agreement was assessed, and any discrepancies were resolved through consensus. Regions of interest (ROIs) were manually drawn on the largest tumor cross-section on the DWI image, excluding areas of cystic degeneration, necrosis, and vessels. T2-weighted and contrast-enhanced images ensured accurate lesion inclusion. The ROIs were automatically transferred to the ADC, *D*, f, *D*^∗^, DDC, and α parametric maps. The mean values of all DWI parameters within each ROI were calculated, with the average across three ROIs used for analysis. The mean ROI area was 1 cm² (range: 0.50–1.78 cm²) (Fig. 2).

### Clinicopathological findings

The clinicopathological parameters included in this study are detailed in Table 1. Tumor staging followed the 8th edition of the AJCC Cancer Staging Manual [10]. Surgical margins were classified as R0 if the tumor was more than 1 mm from the margin microscopically and R1 if tumor cells were visible within 1 mm of the margin. The cut-off value for CA19-9 was set at 39 U/mL, based on standard levels used by our institution. Cut-off values for other continuous variables were determined using X-tile 3.6.1 software.

**Table 1:** Comparison of clinicopathological and DWI Data between two cohorts

Clinicopathological and DWI data	Training cohort *N* = 164 (*N*, %)	Validation cohort *N* = 70 (*N*, %)	*p* value
Age (years)	64 ± 10	62 ± 9	0.239
Sex			
Male	89 (54.3%)	42 (60.0%)	0.419
Female	75 (45.7%)	28 (40.0%)	
CA19-9 (U/mL)			
Normal (≤ 39)	31 (18.9%)	8 (11.4%)	0.184
Elevated (> 39)	133 (81.1%)	62 (88.5%)	
Postoperative 7th day CA19-9 (U/mL)			
Normal (≤ 39)	86 (52.4%)	29 (41.4%)	0.123
Elevated (> 39)	78 (47.6%)	41 (58.5%)	
Tumor location			
Head	108 (64.6%)	53 (78.5%)	0.166
Body and tail	56 (35.4%)	17 (21.4%)	
Type of operation			
PD	102 (62.2%)	53 (75.7%)	0.084
DP	54 (32.9%)	13 (18.6%)	
TP	8 (4.9%)	4 (5.7%)	
T stage			
T1	35 (21.3%)	11 (15.7%)	0.350
T2	96 (58.5%)	48 (68.6%)	
T3	33 (20.1%)	11 (15.7%)	
N stage			
N0	101 (61.6%)	34 (48.6%)	0.182
N1	49 (29.9%)	28 (40.0%)	
N2	14 (8.5%)	8 (11.4%)	
AJCC stage			
IA	25 (15.2%)	8 (11.4%)	0.177
IB	58 (35.4%)	24 (34.3%)	
II A	18 (11.0%)	2 (2.9%)	
II B	48 (29.3%)	28 (40.0%)	
III	15 (9.1%)	8 (11.4%)	
Differentiation			
Poorly	91 (55.5%)	38 (54.3%)	0.891
Intermediate	66 (40.2%)	28 (40.0%)	
Well	7 (4.3%)	4 (5.7%)	
Margin			
R0	114 (69.5%)	45 (64.3%)	0.433
R1	50 (30.5%)	25 (35.7%)	
Nerve invasion			
(+)	138 (84.1%)	59 (84.3%)	0.979
(−)	26 (15.9%)	11 (15.7%)	
Adjuvant chemotherapy			
Yes	86 (52.4%)	43 (61.4%)	0.206
No	78 (47.6%)	27 (38.6%)	
ADC (×10^−^^3^ mm^2^/s)	1.38 ± 0.20	1.37 ± 0.23	0.722
*D* (×10^−3^ mm^2^/s)	1.10 ± 0.26	1.08 ± 0.23	0.625
D^*^ (×10^−3^ mm^2^/s)	14.91 ± 11.07	12.79 ± 8.50	0.154
*f*	0.29 ± 0.10	0.31 ± 0.11	0.227
DDC (×10 ^−3^ mm^2^/s)	1.45 ± 0.95	1.51 ± 1.39	0.704
α	0.77 ± 0.12	0.75 ± 0.11	0.308

*PD* pancreaticoduodenectomy, *DP* distal pancreatectomy, *TP* total pancreatectomy, *CA19-9* carbohydrate antigen 19-9, *ADC* apparent diffusion coefficient, *D* pure diffusion coefficient, D^*^ perfusion-related diffusion coefficient, *f* perfusion fraction, *DDC* distributed diffusion coefficient, *α* stretching coefficient

### Follow-up

Patients underwent regular follow-up visits at our institution post-discharge. For the first 12 months, CA19-9 levels and abdominal CT scans were performed every 3 months, followed by semi-annual evaluations thereafter. Additional imaging studies, including MRI, bone scans, and PET-CT, were conducted as needed. Overall survival (OS) was defined as the time from the date of PDAC diagnosis by MRI until death or the last follow-up date (31 December 2020). Recurrence-free survival (RFS) was defined as the period from surgical resection to tumor recurrence. If no recurrence occurred, the RFS phase ended at death or the last follow-up. Disease progression or recurrence was determined through a combination of imaging and serological exams or surgical exploration. Post-surgical chemotherapy regimens were evaluated by specialists, and patients were monitored regularly.

### Statistical analysis

Categorical variables were compared using the Chi-square test or Fisher’s exact test, while continuous variables were assessed with the *t*-test or Mann–Whitney *U*-test. A significance level of *p* < 0.05 was considered statistically significant. This study employed 101 ML algorithms to develop predictive models, including random survival forest (RSF), elastic network (Enet), Lasso, Ridge, stepwise Cox, CoxBoost, partial least squares regression for Cox (plsRcox), supervised principal components (SuperPC), generalized boosted regression modeling (GBM), and survival support vector machine (survival-SVM). Model performance was evaluated using the concordance index (*C*-index), integrated cumulative/dynamic area under the curve (C/D AUC), and integrated Brier score. The *C*-index, which measures model discrimination, was considered indicative of practical utility if greater than 0.7 higher *C*-index values are associated with better prediction performance. The Brier score assessed predictive accuracy, with values below 0.25 indicating practical applicability; lower Brier scores correspond to improved accuracy. Performance over time was visualized through curves depicting changes in *C*-index and Brier score.

To enhance the interpretability of ML models, global explanations were provided through time-dependent variable importance, partial dependence survival plots, and aggregated survival SHapley Additive exPlanations (SurvSHAP) plots [11]. Subsequently, the focus shifted to localized explanations for individual statistical units, specifically single patients, derived from the examination of SurvSHAP and survival local interpretable model-agnostic explanations (SurvLIME) plots [12]. The statistical analyses were conducted using the R version 4.2.1 software.

## Results

### Clinicopathological and DWI data characteristics

The study included 234 patients with PDAC who underwent R0/R1 radical resection. The median follow-up duration was 22 months. By the end of follow-up, 169 patients (72.2%) had died, and 177 patients (75.6%) experienced postoperative recurrence. Clinical characteristics and DWI data showed no significant disparity between the training (*n* = 164) and validation (*n* = 70) cohorts (Table 1) and two centers (Table S1).

### Development and comparison of the ML model

**Fig. 3:**
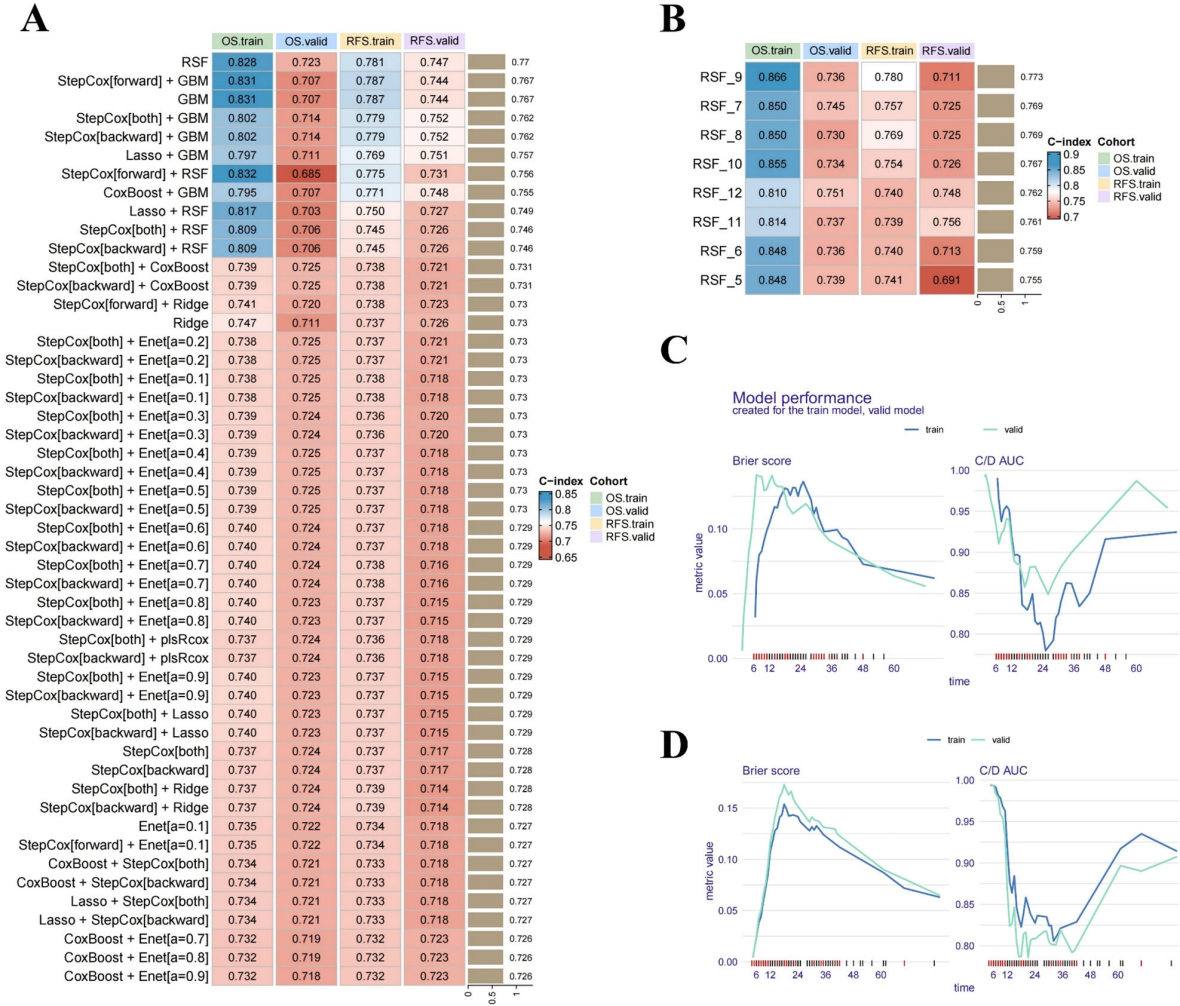
Prediction models were developed using 101 ML algorithms, and the best-performing model was identified through evaluation. **A** Prediction models developed using 101 ML algorithms were evaluated for their performance in predicting OS and RFS in both the training and validation cohorts using the key metric, the *C*-index. The models were ranked based on the average *C*-index across cohorts, identifying the random survival forest model as the best-performing ML model for predictive capability. **B** The random survival forest model was constructed using the top 5–12 variables ranked by average *C*-index (OS train, OS valid, RFS train, and RFS valid cohorts). It was observed that when the top nine variables were included, the average *C*-index across the four cohorts was the highest, indicating the best predictive performance of the model. In both the training and validation cohorts, the random survival forest model, identified as the best-performing model, achieved Brier scores below 0.13 and C/D AUC curves exceeding 0.85 for predicting OS (**C**) and RFS (**D**) following radical resection of PDAC, demonstrating remarkable predictive accuracy

In the training cohort, we constructed an ML framework including 10 individual ML algorithms and 101 algorithm combinations to predict postoperative OS and RFS in PDAC. The *C*-index for each model was calculated in both cohorts to evaluate predictive performance. The results showed the random survival forest model achieved the highest *C*-index for predicting both postoperative OS and RFS (best model). The *C*-index in the training cohort was 0.828 for OS 0.781 for RFS, 0.723 for OS, and 0.747 for RFS in the validation cohort. The results of the top 50 models ranked by *C*-index are shown in Fig. 3A, while the *C*-index results for all 101 models can be found in Figure S2. To achieve the optimal balance between predictive performance and the number of variables, we developed models for four cohorts using the RSF model with 5–12 top variables included. The results showed the random survival forest model with nine key variables (*D* value, T-stage, ADC-value, postoperative 7th day CA19-9 level, AJCC stage, tumor differentiation, type of operation, tumor location, and age) showed the best *C*-index (Fig. 3B). The model’s error rate stabilized with 600 trees (Fig. S3).

To further evaluate the time-dependent predictive performance of the best model, we assessed Brier scores and cumulative/dynamic AUC values for the best model predicting OS and RFS in both cohorts. As shown in Fig. 3C, D, the Brier scores for predicting OS and RFS were below 0.13 in both cohorts, demonstrating significant predictive accuracy. The C/D AUC curves also indicated excellent predictive capability, with values exceeding 0.85 for both OS and RFS in both cohorts. Additionally, ROC curves (Fig. S4) for the random survival forest model showed high performance: the AUC for OS was 0.943, 0.941, and 0.943 at 1 year, 2 years, and 3 years in the training cohort, and 0.892, 0.915, and 0.939 in the validation cohort. For RFS, the AUC values were 0.915, 0.924, and 0.955 at 1, 2, and 3 years in the raining cohort, and 0.922, 0.972, and 0.989 in the validation cohort. These results indicate the model demonstrates a strong goodness of fit for predicting both OS and RFS.

### Explanation of the ML model

**Fig. 4:**
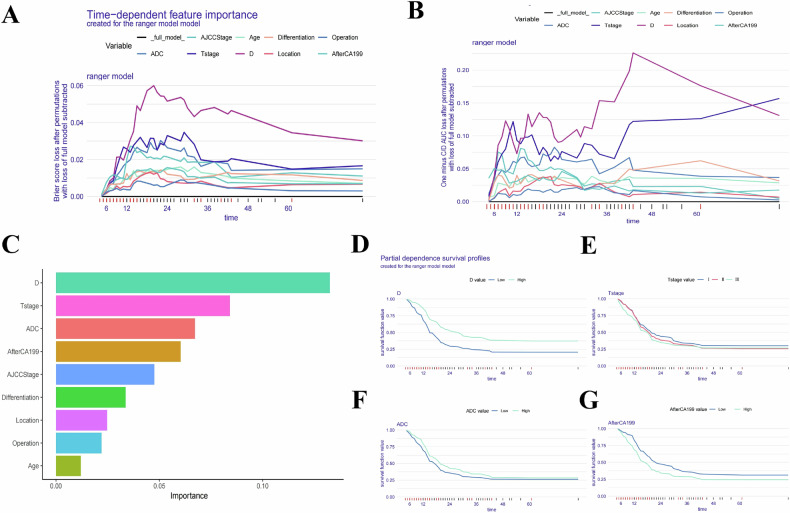
Evaluation and explanation of the global and localized impact of the random survival forest model in predicting OS. **A**, **B** The global impact of each variable on the random survival forest model’s predictions for OS was assessed using Brier score loss and C/D AUC loss after permutation. The analyses revealed that variable importance evolves over time, with the Brier score and C/D AUC loss demonstrating a pronounced time-dependent effect. Notably, the *D* value consistently showed the greatest significance as survival time increased. **C** The variable importance summary plot ranks nine key features by their influence on OS. **D**–**G** Partial dependence survival profiles (PDPs) illustrate how changes in individual variables, such as *D* value, T stage, ADC value, and postoperative 7th day CA19-9 level, affect OS while holding other variables constant. Patients with lower *D* value experienced a more rapid decline in survival compared to those with higher *D* value, reflecting the significant protective impact of *D* value on postoperative survival. Narrow bands in the PDP plots indicate stable predictions, while wider bands suggest greater sensitivity to variable changes

**Fig. 5:**
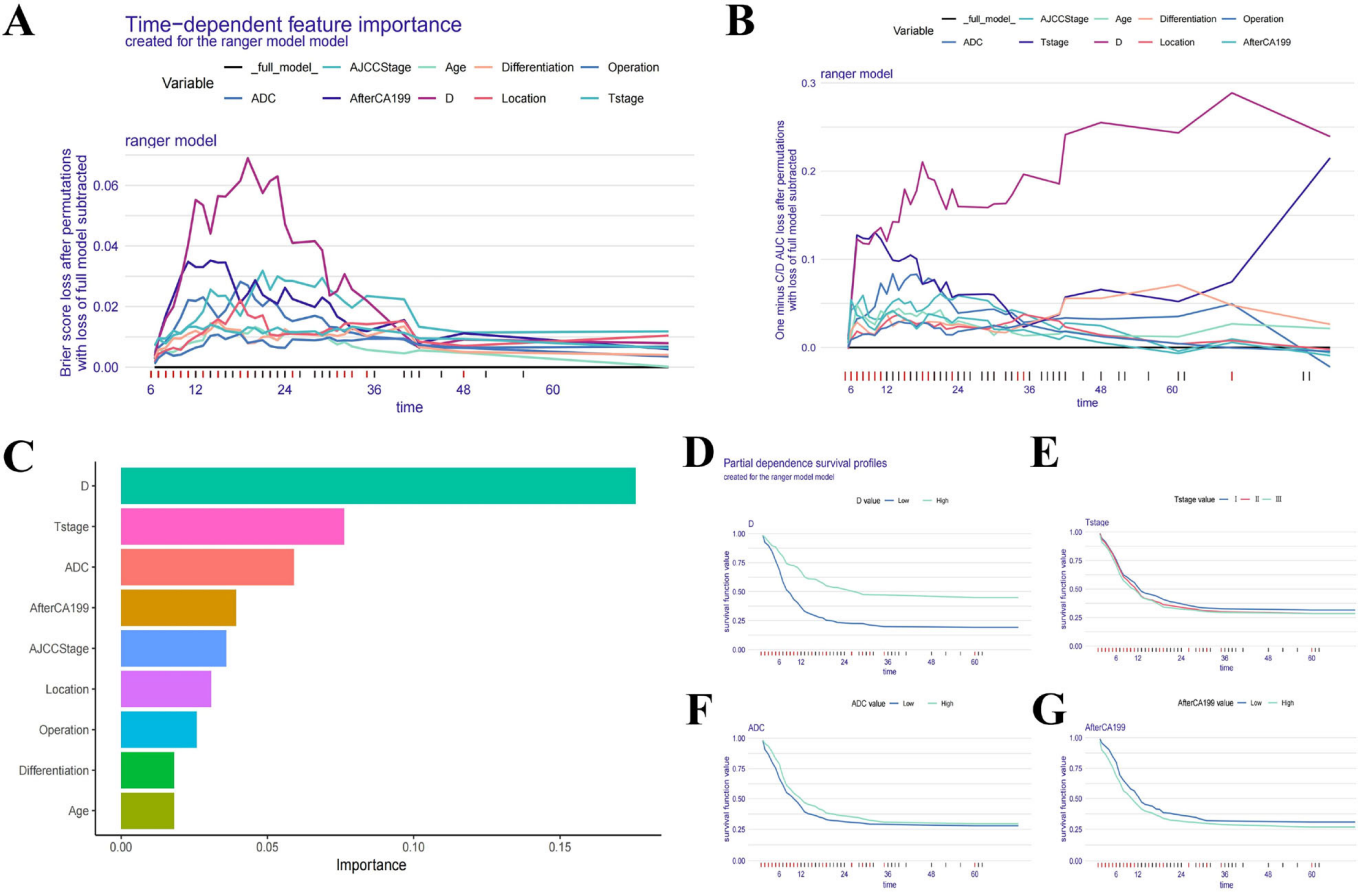
Evaluation and explanation of the global and localized impact of the random survival forest model in predicting RFS. **A**, **B** The global impact of each variable on the random survival forest model’s predictions for RFS was evaluated using Brier score loss and C/D AUC loss after permutation. Similar to OS, variable importance demonstrated a time-dependent effect, with the *D* value consistently identified as the most significant variable for predicting RFS over time. **C** The variable importance summary plot ranks nine key features by their influence on RFS. **D**–**G** Partial dependence survival profiles (PDPs) highlight how individual variables, such as *D* value, T stage, ADC value, and postoperative 7th day CA19-9 level, influence RFS predictions while keeping other variables constant. Patients with lower *D* values showed a more rapid decline in RFS compared to those with higher *D* values. The PDP plots show narrow and overlapping bands for certain variables, suggesting stable predictions, while wider bands indicate that even small changes in the variable values can significantly affect RFS predictions

In our study, we assessed the global impact of each variable on the model’s predictions using two main approaches: Brier score loss and C/D AUC loss after permutation (Figs. 4 and 5A, B). These analyses revealed variable importance evolves over time, with the Brier score and C/D AUC loss showing a more pronounced time-dependent effect. Notably, the *D* value consistently demonstrated the greatest significance for predicting both OS and RFS as survival time increased.

The variable importance summary plot (Figs. 4C and 5C) ranks 9 key features by their influence on OS and RFS, with their importance values and rankings detailed in Table 2 for OS and Table 3 for RFS. The SurvSHAP bee swarm plot (Fig. S5) further illustrates the global impact of these features. Higher *D* values (> 1.18 × 10^−3^ mm^2^/s), lower T stages, higher ADC values (> 1.33 × 10^−3^ mm^2^/s), lower postoperative 7th day CA19-9 level, lower AJCC stages, better tumor differentiation, pancreaticoduodenectomy, and age ≥ 65 years were associated with reduced risk of poor postoperative outcomes and recurrence.

**Table 2:** The importance values and ranking table of variables included in the random survival forest model for predicting postoperative OS

Variable	Importance	Relative importance	Rank
*D* value	0.145	1	1
T stage	0.081	0.559	2
ADC value	0.071	0.490	3
Postoperative 7th day CA19-9 level	0.062	0.428	4
AJCC stage	0.045	0.310	5
Tumor differentiation	0.036	0.248	6
Tumor location	0.023	0.159	7
Type of operation	0.020	0.138	8
Age	0.009	0.062	9

**Table 3:** The importance values and ranking table of variables included in the random survival forest model for predicting postoperative RFS

Variable	Importance	Relative importance	Rank
*D* value	0.172	1	1
T stage	0.076	0.442	2
ADC value	0.063	0.366	3
Postoperative 7th day CA19-9 level	0.045	0.262	4
AJCC stage	0.042	0.244	5
Tumor location	0.032	0.186	6
Type of operation	0.026	0.151	7
Tumor differentiation	0.021	0.122	8
Age	0.020	0.116	9

Partial dependence survival profiles (PDP) offer additional insights by showing how changes in individual variables affect OS and RFS while holding other variables constant. Narrow and overlapping bands in PDP plots suggest that predictions are relatively stable despite changes in variable values. In contrast, wider bands indicate even small changes in variables, which also can significantly impact predictions. Specifically, patients with low *D* value experienced a more rapid decline in survival compared to those with high *D* value (Figs. 4D–G and 5D–G).

### Assessment and risk stratification of the ML model

**Fig. 6:**
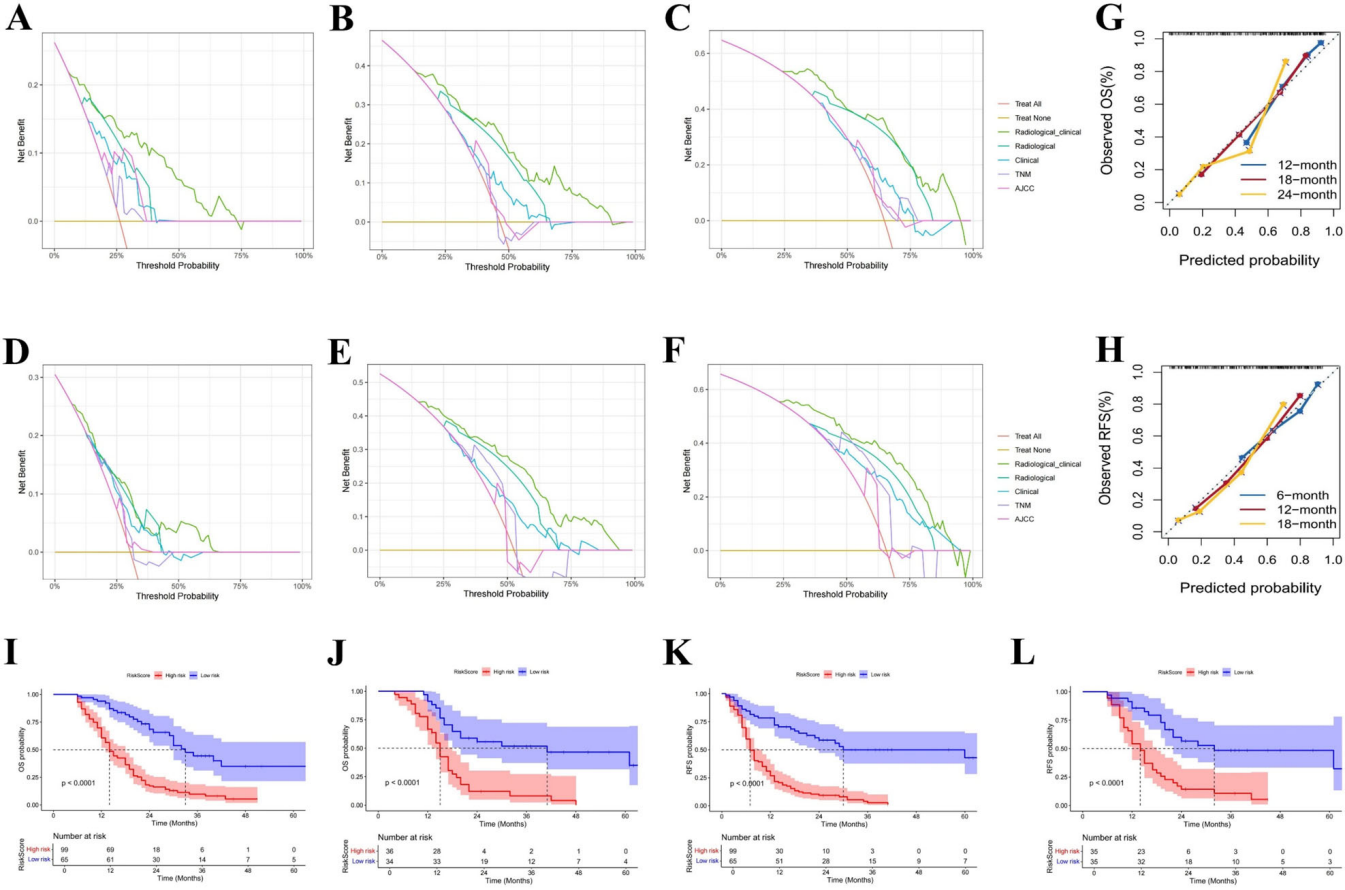
Performance evaluation of the radiological–clinical random survival forest model for predicting OS and RFS. **A**–**C** DCA comparing the radiological–clinical random survival forest model to four common models (radiological, clinical, TNM, and AJCC stage) for predicting OS in the training cohort at 12 months (**A**), 18 months (**B**), and 24 months (**C**). **D**–**F** DCA for the radiological–clinical model and the four common models for predicting RFS in the training cohort at 6 months (**D**), 12 months (**E**), and 18 months (**F**). The radiological–clinical model consistently outperformed the other models, demonstrating superior clinical utility. **G**, **H** Calibration curves for OS (**G**) and RFS (**H**) in the training cohort at corresponding time points (12 months, 18 months, 24 months for OS and 6 months, 12 months, 18 months for RFS). The curves align closely with the diagonal dashed line, indicating strong agreement between predicted and observed outcomes. **I**–**L** Kaplan–Meier survival curves stratifying patients into low-risk and high-risk groups for OS in the training cohort (**I**) and validation cohort (**J**), and for RFS in the training cohort (**K**) and validation cohort (**L**). The model effectively separated patients into distinct risk categories, with significant survival differences (*p* < 0.0001)

The performance of the ML models was thoroughly evaluated using cohort validation, calibration curves, and decision curve analysis (DCA). The radiological–clinical random survival forest model was compared against other common models, including the TNM stage, AJCC stage, and radiological and clinical models. The DCA results demonstrated the radiological–clinical model consistently outperformed these alternatives in both training and validation cohorts, highlighting its superior predictive ability and clinical benefit (Figs. 6A–F and S6). In addition, internal validation was conducted using the Bootstrap method with 1000 repetitions. Calibration curves for predicting postoperative OS at 12 months, 18 months, and 24 months, as well as RFS at 6 months, 12 months, and 18 months, closely aligned with the diagonal dashed line in both training and validation cohorts, indicating strong consistency between predicted and observed outcomes (Figs. 6G, H and S6).

Survival and recurrence risk scores were derived from the optimal model, with cut-off points determined using X-tile software. Patients were categorized into low-risk (≤ 16.73) and high-risk (> 16.73) groups for OS, and low-risk (≤ 29.05) and high-risk (> 29.05) groups for RFS. K-M survival curves revealed that the model effectively stratified patients into distinct risk categories for both OS and RFS following radical resection of PDAC (*p* < 0.0001), underscoring its utility in guiding individualized treatment (Figs. 6I–L and S7).

## Discussion

This study, based on preoperative multiparametric quantitative DWI and clinical-pathological data, has for the first time developed and validated a radiological–clinical predictive model using the most comprehensive set of 101 ML algorithms. This model is designed to assess survival and recurrence risks following curative resection of PDAC. Cohort validation, *C*-index, Brier score, C/D AUC value, DCA curves, and concordance curves all indicate that the random survival forest model, constructed with nine key features, exhibits the best predictive performance and risk stratification capabilities, outperforming other traditional models. Interpretation of the random survival forest model shows the *D* value and ADC value from preoperative multiparametric quantitative DWI are among the top three predictive variables, highlighting their significant role. Therefore, it plays an indispensable role in predicting outcomes following direct radical surgery for PDAC. The findings of this study, and the developed radiological–clinical predictive model, will aid clinicians in effectively assessing risk for patients after surgery, guiding the formulation of more comprehensive treatment plans and early intervention strategies to reduce the risk of recurrence and metastasis in PDAC.

In the importance analysis of the random survival forest model, the *D* value was identified as the most significant predictive factor. Previous research indicates the *D* value reflects diffusion related to the microscopic structure of tumor tissue, excluding the influence of vascular perfusion. Studies conducted at our center also found the *D* value is negatively correlated with cancer-associated dense fibrosis and tumor tissue hypoxia. A low *D* value suggests extensive and dense collagen fibers markedly restrict water molecule diffusion, leading to severe tissue hypoxia [13, 14]. Additionally, fibrosis can promote local invasion through fibroblasts [15], thereby increasing cancer malignancy [16, 17]. Hypoxia is a critical feature of PDAC [18] and is associated with both the malignancy and invasiveness of tumors [19]. A preliminary study by Klaassen et al also showed similar survival analysis results [9]. These above results highlight the significant predictive value of the *D* value for PDAC prognosis following direct radical surgery resection, with lower *D* values correlating with increased risks of survival and recurrence.

In addition to the *D* value, the ADC value also plays a crucial role. The ADC value is a quantitative parameter for evaluating the diffusion of water molecules [20, 21]. Previous studies in our center have found the ADC value is also negatively correlated with the degree of cancer-related dense fibrosis and tumor tissue hypoxia, but the degree is significantly lower than the *D* value. This indicates the ADC value is also affected by dense fibrosis, and abundant and dense collagen fibers hinder the diffusion of water molecules [22], which is consistent with the findings of other studies [23, 24]. Previous studies have reported the value of ADC in the prognosis of PDAC [25, 26]. However, the ADC value cannot accurately reflect the true diffusion motion of water molecules in tumor tissues, which is susceptible to various factors such as tumor cell number, collagen fibers, and vascular density [27, 28]. Thus, while the preoperative ADC value is a significant risk factor for postoperative survival and recurrence, the *D* value appears to have a stronger predictive significance. Nonetheless, the ADC value, as a reflection of the overall pancreatic cancer tumor microenvironment, also retains significant predictive value that should not be overlooked.

Previous studies have indicated the perfusion fraction (*f* value) derived from multiparameter quantitative DWI is associated with vascular distribution in PDAC and can be used to assess tumor blood perfusion levels [29]. However, the consistency of the *f* value measurement is less stable compared to the ADC and *D* values and is influenced by the choice of B-value in MRI [14]. This variability may contribute to the uncertainty in *f* value predictions. As for other quantitative indicators, there is currently no literature reporting their correlation with prognosis. Future research is needed to further explore the predictive value of these indicators.

This study also found clinical factors such as tumor T and AJCC stage [30, 31], postoperative 7th day CA19-9 level [32], tumor differentiation [33], type of operation with tumor location [34] and age [35] are associated with postoperative survival and recurrence, which is consistent with most previous studies [36–38]. Currently, most models for predicting the prognosis of PDAC are also based on these indicators. Additionally, this study compared the predictive performance of the radiological–clinical model with models based on the TNM stage, AJCC stage, and those using only clinical data or DWI quantitative parameters. The results demonstrated the random survival forest model offers superior predictive capability and the greatest clinical benefit. This highlights the effectiveness of the radiological–clinical prediction model established in this study and underscores the significant value of preoperative multiparametric quantitative DWI in predicting survival and recurrence risk following radical resection for PDAC. In addition, there have been relevant research reports on the effective prediction of PDAC prognosis through MRI radiomics [39], but this method of obtaining quantitative parameters through high-throughput analysis cannot fully meet the needs of clinical practicality, which is not conducive to clinicians proficiently and rapidly mastering the method. At the same time, the quantitative parameters obtained from MRI radiomics cannot further explain the clinical pathological significance.

However, this study has several limitations. Firstly, being a retrospective study, it may be subject to selection bias. Future prospective validation studies or multicenter collaborations are anticipated to enhance the generalizability of the research findings. Secondly, the MRI parameter settings between the two centers may not be entirely consistent, potentially affecting the results. Nevertheless, cohort validation and evaluation indicate the prediction model demonstrates good extrapolation ability and stability. Thirdly, because MRI images were acquired during free breathing, the multiparameter quantitative DWI parameters may be influenced by motion-induced variability. However, given that the pancreas is situated in the retroperitoneum, the impact of respiratory motion is likely less pronounced compared to other abdominal organs [40].

## Conclusion

This study highlights the significant potential of multiparametric quantitative DWI in evaluating the prognosis of PDAC patients. The random survival forest ML model, which integrates preoperative multiparametric quantitative DWI with clinical features, exhibited robust predictive performance and effective risk stratification for survival and recurrence following radical resection of PDAC. This model is anticipated to become increasingly vital in guiding personalized precision treatment for PDAC in the future.

## Supplementary information

ELECTRONIC SUPPLEMENTARY MATERIAL

## Abbreviations

ADC: Apparent diffusion coefficient
AUC: Area under the curve
DCA: Decision curve analysis
DWI: Diffusion-weighted imaging
ML: Machine learning
OS: Overall survival
PDAC: Pancreatic ductal adenocarcinoma
RFS: Recurrence-free survival
ROI: Region of interest
