# Supplementary material for: A machine learning model based on preoperative multiparametric quantitative DWI can effectively predict the survival and recurrence risk of pancreatic ductal adenocarcinoma
Source: Insights Imaging. 2025 Feb 17;16:38. doi: 10.1186/s13244-025-01915-9 (PMC11833029; doi:10.1186/s13244-025-01915-9)
Supplement: Supplementary file 1 — ELECTRONIC SUPPLEMENTARY MATERIAL [file 13244_2025_1915_MOESM1_ESM.pdf]

A machine learning model based on preoperative multiparametric quantitative DWI can effectively predict the survival and recurrence risk of pancreatic ductal adenocarcinoma

ELECTRONIC SUPPLEMENTARY MATERIAL

Methods

Magnetic resonance imaging techniques

MRI scans in the main study cohort were obtained using the GE Discovery MR750 3.0T MRI scanner and 8-channel abdominal coil. The MRI protocol consisted of the axial T2-weighted image (repetition time/echo time (TR/TE), 12000-15000/81 msec; slice thickness, 6.0 mm; intersection gap, 1 mm; field of view, 32×32 cm; matrix, 320×320) and axial T1-weighted LAVA images (TR/TE 4/2.4 msec; slice thickness, 5.0 mm; field of view, 36×36 cm; matrix, 264×56). DWI was performed with an axial, free-breathing, single-shot echo-planar imaging with the following parameters: TR/TE, 15000/70 msec; slice thickness, 6.0 mm; intersection gap, 1.0 mm; bandwidth, 250 kHz; field of view, 35×35 cm; matrix, 160×160; parallel imaging factor of 2; 8 b values (0, 25, 50, 100, 200, 500, 800, 1000 s/mm^2).The IVIM sequence takes 2 minutes and 3 seconds.

MRI scans in the external validation cohort were obtained using the GE Discovery MR750 3.0T MRI scanner and 8-channel abdominal coil. The MRI protocol consisted of the axial T2-weighted image (repetition time/echo time (TR/TE), 15000/85 msec; slice thickness, 4.0 mm; intersection gap, 1 mm; field of view, 28×28 cm; matrix, 320×224) and axial T1-weighted LAVA images (TR/TE 4/min msec; slice thickness, 6.0 mm; field of view, 34×34 cm; matrix, 320×224). DWI was performed with an axial, free-breathing, single-shot echo-planar imaging with the following parameters: TR/TE, 15000/60 msec; slice thickness, 6.0 mm; intersection gap, 1.0 mm; bandwidth, 250 kHz; field of view, 34×34 cm; matrix, 128×160; parallel imaging factor of 2; 8 b values

(0，50，100，150，200，500，800，1000 s/mm^2). The IVIM sequence takes 6 minutes and 15 seconds.

ADC was automatically calculated using the mono-exponential model with all b values:

$S(b) = S0 \cdot \exp(-b \cdot ADC)$ (1)

The bi-exponential model, the mathematical relationship between b values and signal intensities, could be described using the following formula:

$S(b) = [(1-f) \cdot \exp(-b \cdot D) + f \cdot \exp(-b \cdot D^*)]$ (2)

where D indicates unalloyed water molecular diffusion, and f and D* are linked to the microcapillary perfusion effect, which may influence signal attenuation at low b values (b < 200 s/mm^2). However, at high b values, the perfusion-related contribution to the signal attenuation becomes negligible; hence, true diffusion could be detected.

Finally, by fitting the stretched-exponential model, DDC and α were calculated as follows:

$S(b) = S0 \cdot \exp(-b \cdot DDC\alpha)$ (3)

where α varies from zero to one and characterizes the deviation of the signal decay from a mono-exponential decay. DDC indicates the composite ADC that is weighted by the volumes of water molecules with different diffusion coefficients.

Logrank Tests

Numeric Results for the Logrank Test in Terms of Sample Size

Solve For: Sample Size
Alternative Hypothesis: One-Sided

Power	N1	N2	N	Haz Ratio HR	Ctrl Med Surv Time M1	Trt Med Surv Time M2	Acc-rual Pat'n	Acc-rual Time/ Total Time	Ctrl Loss	Trt Loss	Ctrl to Trt	Trt to Ctrl	Alpha	Beta
0.80136	72	48	120	2.35714	33	14	Equal	3 / 8	0.05	0.05	0	0	0.15	0.19864

Power	The probability of rejecting a false null hypothesis when the alternative hypothesis is true.
N1|N2|N	The sample sizes of the control group, treatment group, and both groups, respectively.
HR	Hazard Ratio. The controls group's median survival time divided by the treatment group's median survival time.
Median Survival Time	The time until half the subjects fail.
Accrual Time	The number of time periods (years or months) during which accrual takes place.

Figure S1. According to the sample size estimation based on the study design, including more than 120 cases in the study was sufficient to effectively achieve the expected research outcomes.

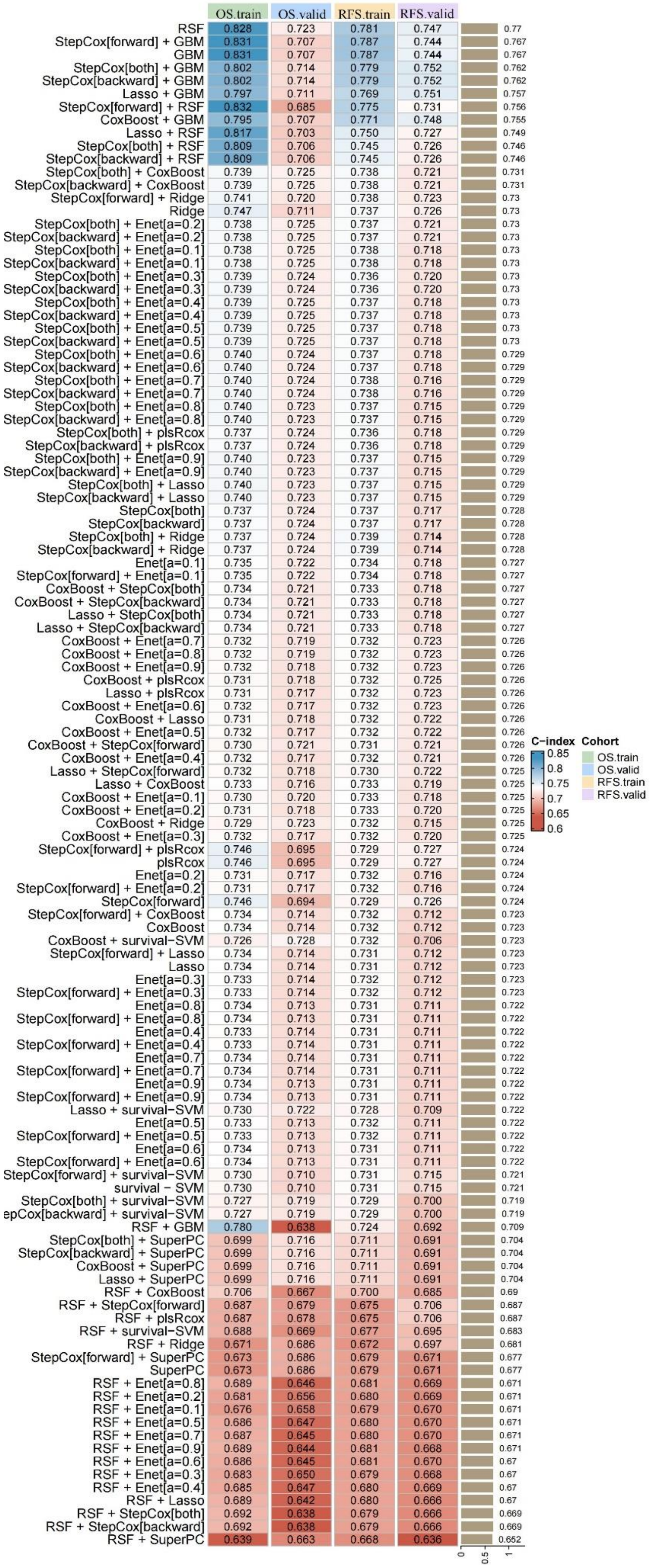

Figure S2. The C-index results for all 101 models in both the training and validation cohorts identifying the random survival forest model as the best-performing machine learning model for predictive capability.

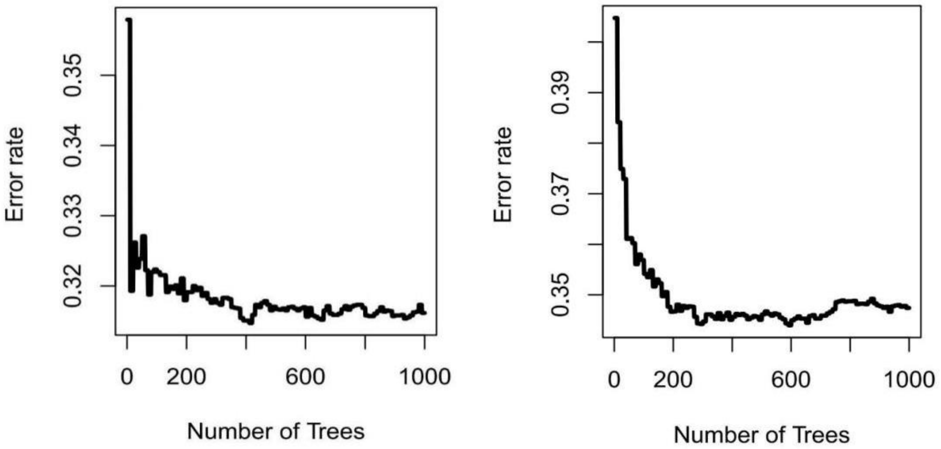

Figure S3. (A) Error rate of OS prediction after direct radical resection of PDAC using random survival forest; **(B)** Error rate of RFS prediction after direct radical resection of PDAC using random survival forest. The model's error rate stabilized with 600 trees.

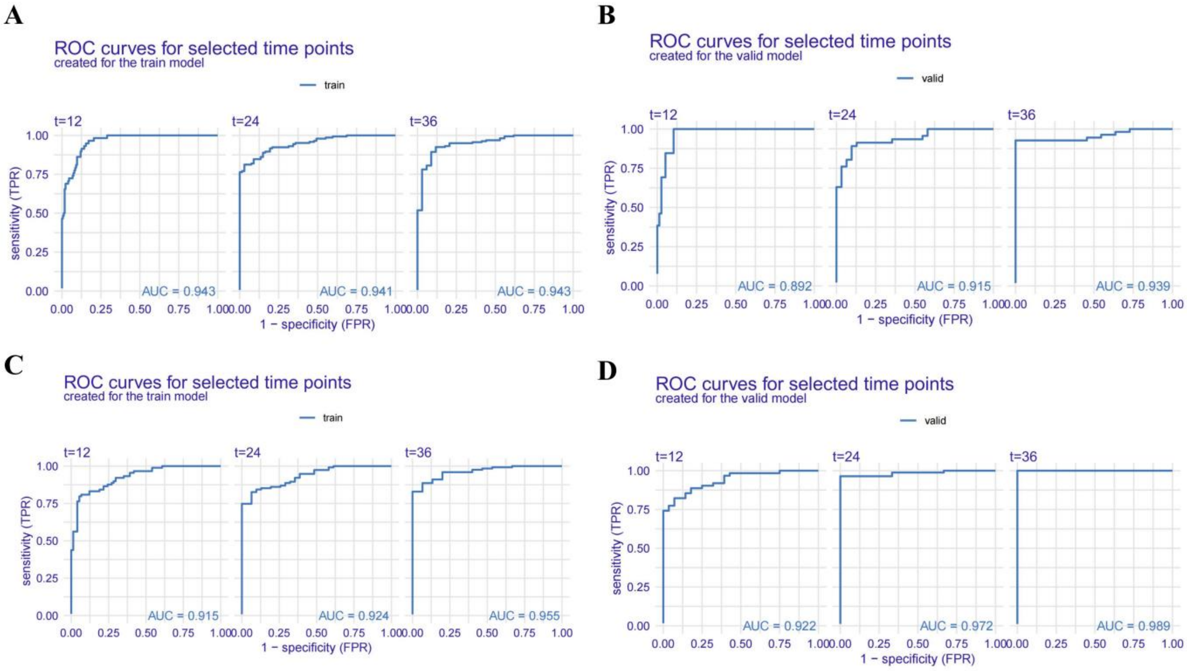

Figure S4. (A) ROC curve analysis of the ranger OS model assessing the accuracy of 1-, 2-, and 3-year predictions for training cohort. **(B)** ROC curve analysis of the ranger OS model assessing the accuracy of 1-, 2-, and 3-year predictions for validation cohort. **(C)** ROC curve analysis of the ranger RFS model assessing the accuracy of 1-, 2-, and 3-year predictions for training cohort.**(D)** ROC curve analysis of the ranger RFS model assessing the accuracy of 1-, 2-, and 3-year predictions for validation cohort.

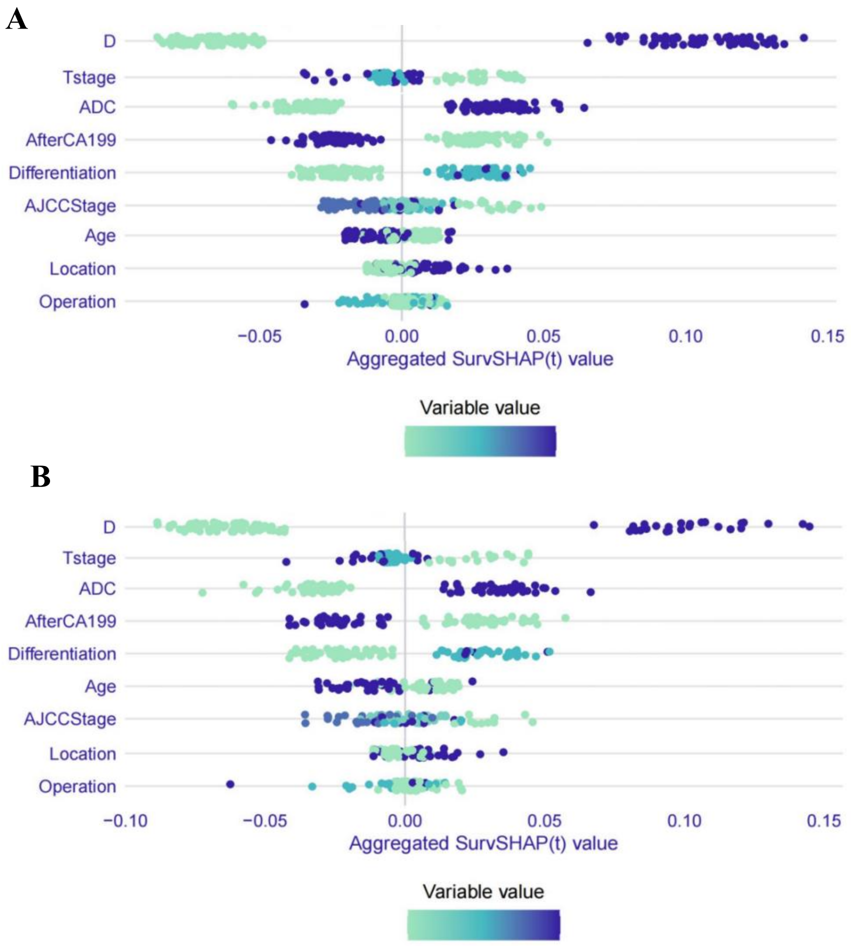

Figure S5. (A) The SurvSHAP summary plot illustrating the global impact of features in the OS random survival forest model. Each point on the bee swarm plot represents a specific feature for an individual patient. The y-coordinate denotes the feature, while the x-coordinate represents its impact on the model output. The color of each point reflects the feature value, ranging from high to low, as indicated by the color bar below. Features on the y-axis are ordered by their significance.**(B)** The SurvSHAP summary plot illustrating the global impact of features in the RFS random survival forest model, presented in the same format as (A).

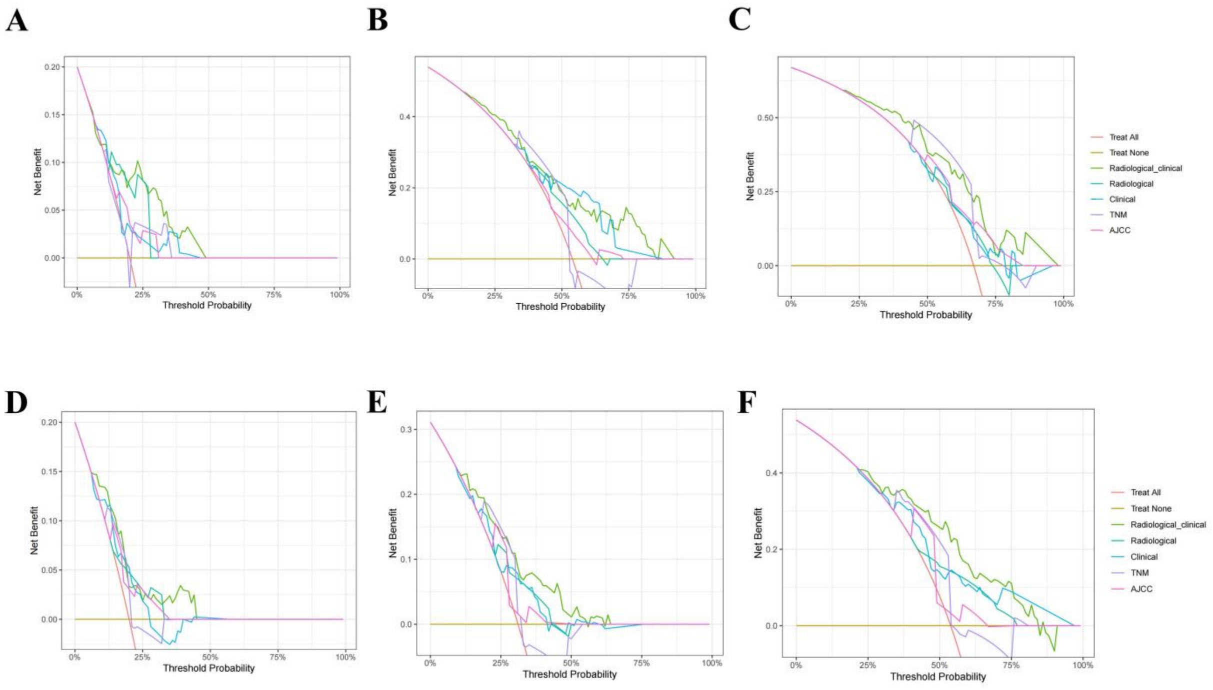

Figure S6. (A-C) Decision curve analysis (DCA) comparing the radiological-clinical random survival forest model to four common models (radiological, clinical, TNM, and AJCC stage) for predicting OS in the validation cohort at 12 months (A), 18 months (B), and 24 months (C). **(D-F)** DCA for the radiological-clinical model and the four common models for predicting RFS in the validation cohort at 6 months (D), 12 months (E), and 18 months (F). The radiological-clinical model consistently outperformed the other models, demonstrating superior clinical utility.

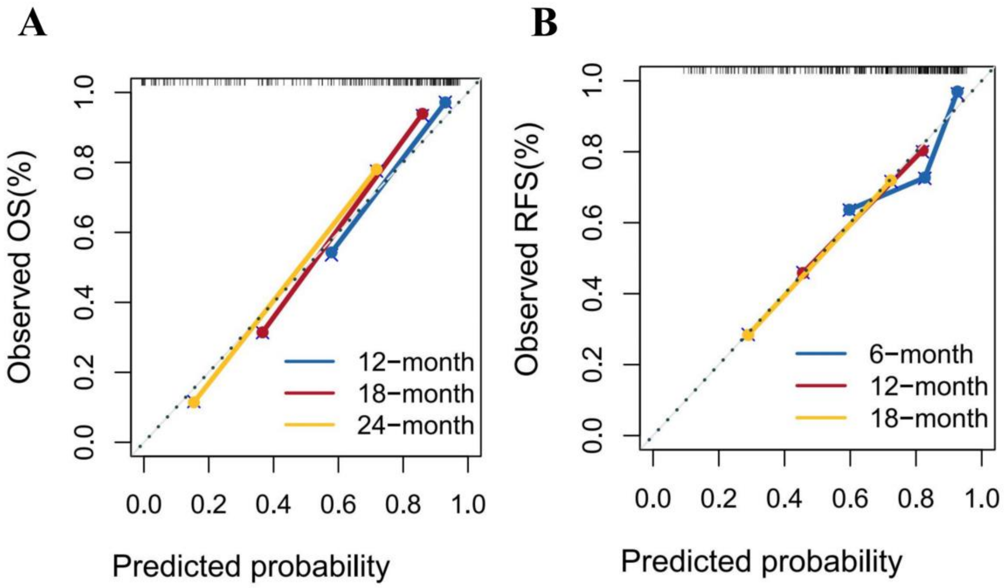

Figure S7. Calibration curves for OS **(A)** and RFS **(B)** in the validation cohort at corresponding time points (12, 18, 24 months for OS and 6, 12, 18 months for RFS). The curves align closely with the diagonal dashed line, indicating strong agreement between predicted and observed outcomes.

Table S1. Comparison of clinicopathological and DWI Data between two centers.

Clinicopathological and DWI Data	Center 1<br>N=136（N,%）	Center 2<br>N=98（N,%）	*P* value
Age (years)	64 ± 9	63 ± 11	0.455
Sex			
Male	74（54.4%）	57（58.2%）	0.568
Female	62（45.5%）	41（41.8%）	
CA19-9（U/ml）			
Normal（≤39）	22（16.2%）	17（17.3%）	0.813
Elevated（＞39）	114（83.8%）	81（83.7%）	
Postoperative 7th day CA19-9（U/ml）			
Normal（≤39）	69（50.7%）	46（46.9%）	0.567
Elevated（＞39）	67（49.3%）	52（53.1%）	
**Tumor location**			
**Head**	90（66.2%）	71（72.4%）	0.307
**Body and Tail**	46（33.8%）	27（27.6%）	
**Type of operation**			
**PD**	86（63.2%）	69（70.4%）	0.502
**DP**	42（30.9%）	25（25.5%）	
**TP**	8（5.9%）	4（4.1%）	
T **stage**			
T1	22（16.2%）	24（24.5%）	0.259
T2	86（63.2%）	58（59.2%）	

T3	28（20.6%）	16（16.3%）	
N **stage**			
N0	76（55.9%）	59（60.2%）	0.650
N1	48（35.3%）	29（29.6%）	
N2	12（8.8%）	10（10.2%）	
AJCC **stage**			
IA	15（11.0%）	18（18.4%）	0.433
IB	50（36.8%）	32（32.6%）	
II A	10（7.3%）	10（10.2%）	
II B	48（35.3%）	28（28.6%）	
III	13（9.6%）	10（10.2%）	
Differentiation			
Poorly	71（52.2%）	58（59.2%）	0.498
Intermediate	59（43.4%）	35（35.7%）	
Well	6（4.4%）	5（5.1%）	
**Margin**			
R0	94（69.1%）	65（66.3%）	0.652
R1	42（30.9%）	33（33.7%）	
**Nerve invasion**			
**(+)**	119（87.5%）	78（79.6%）	0.102
**(--)**	17（12.5%）	20（20.4%）	
**Adjuvant Chemotherapy**			
**Yes**	82（60.3%）	47（48.0%）	0.061
**No**	54（41.9%）	51（52.0%）	
ADC（$\times 10^{-3} mm^2/s$）	1.39 ± 0.22	1.37 ± 0.18	0.400
D（$\times 10^{-3} mm^2/s$）	1.11 ± 0.26	1.08 ± 0.24	0.300
D*（$\times 10^{-3} mm^2/s$）	14.51 ± 10.56	13.96 ± 10.16	0.691

Insights Imaging (2025) Qu C, Zeng P, Li C, et al.

f	0.28 ± 0.11	0.30 ± 0.09	0.142
DDC（$\times 10^{-3} mm^2/s$）	1.46 ± 1.03	1.48 ± 1.17	0.872
α	0.77 ± 0.12	0.76 ± 0.11	0.458

PD—pancreaticoduodenectomy; DP—distal pancreatectomy; TP—total pancreatectomy; CA19-9—carbohydrate antigen 19-9; ADC—apparent diffusion coefficient;

D—pure diffusion coefficient; D*—perfusion-related diffusion coefficient; f—perfusion fraction;

DDC—distributed diffusion coefficient; α—stretching coefficient.

TRIPOD+AI

Version: 11-January-2024

Section/Topic	Item	Development / evaluation[1]	Checklist item	Reported on page
**TITLE**				
Title	1	D;E	Identify the study as developing or evaluating the performance of a multivariable prediction model, the target population, and the outcome to be predicted	1
**ABSTRACT**				
Abstract	2	D;E	See TRIPOD+AI for Abstracts checklist	1
**INTRODUCTION**				
Background	3a	D;E	Explain the healthcare context (including whether diagnostic or prognostic) and rationale for developing or evaluating the prediction model, including references to existing models	3
	3b	D;E	Describe the target population and the intended purpose of the prediction model in the context of the care pathway, including its intended users (e.g., healthcare professionals, patients, public)	3
	3c	D;E	Describe any known health inequalities between sociodemographic groups	2
Objectives	4	D;E	Specify the study objectives, including whether the study describes the development or validation of a prediction model (or both)	3
**METHODS**				
Data	5a	D;E	Describe the sources of data separately for the development and evaluation datasets (e.g., randomised trial, cohort, routine care or registry data), the rationale for using these data, and representativeness of the data	3
	5b	D;E	Specify the dates of the collected participant data, including start and end of participant accrual; and, if applicable, end of follow-up	3
Participants	6a	D;E	Specify key elements of the study setting (e.g., primary care, secondary care, general population) including the number and location of centres	4
	6b	D;E	Describe the eligibility criteria for study participants	4
	6c	D;E	Give details of any treatments received, and how they were handled during model development or evaluation, if relevant	4
Data preparation	7	D;E	Describe any data pre-processing and quality checking, including whether this was similar across relevant sociodemographic groups	5
Outcome	8a	D;E	Clearly define the outcome that is being predicted and the time horizon, including how and when assessed, the rationale for choosing this outcome, and whether the method of outcome assessment is consistent across sociodemographic groups	5
	8b	D;E	If outcome assessment requires subjective interpretation, describe the qualifications and demographic characteristics of the outcome assessors	5
	8c	D;E	Report any actions to blind assessment of the outcome to be predicted	5
Predictors	9a	D	Describe the choice of initial predictors (e.g., literature, previous models, all available predictors) and any pre-selection of predictors before model building	5
	9b	D;E	Clearly define all predictors, including how and when they were measured (and any actions to blind assessment of predictors for the outcome and other predictors)	4
	9c	D;E	If predictor measurement requires subjective interpretation, describe the qualifications and demographic characteristics of the predictor assessors	4
Sample size	10	D;E	Explain how the study size was arrived at (separately for development and evaluation), and justify that the study size was sufficient to answer the research question. Include details of any sample size calculation	4
Missing data	11	D;E	Describe how missing data were handled. Provide reasons for omitting any data	4
Analytical methods	12a	D	Describe how the data were used (e.g., for development and evaluation of model performance) in the analysis, including whether the data were partitioned, considering any sample size requirements	5
	12b	D	Depending on the type of model, describe how predictors were handled in the analyses (functional form, rescaling, transformation, or any standardisation).	5
	12c	D	Specify the type of model, rationale[2], all model-building steps, including any hyperparameter tuning, and method for internal validation	5
	12d	D;E	Describe if and how any heterogeneity in estimates of model parameter values and model performance was handled and quantified across clusters (e.g., hospitals, countries). See TRIPOD-Cluster for additional considerations[3]	6
	12e	D;E	Specify all measures and plots used (and their rationale) to evaluate model performance (e.g., discrimination, calibration, clinical utility) and, if relevant, to compare multiple models	6
	12f	E	Describe any model updating (e.g., recalibration) arising from the model evaluation, either overall or for particular sociodemographic groups or settings	6
	12g	E	For model evaluation, describe how the model predictions were calculated (e.g., formula, code, object, application programming interface)	6
Class imbalance	13	D;E	If class imbalance methods were used, state why and how this was done, and any subsequent methods to recalibrate the model or the model predictions	5
Fairness	14	D;E	Describe any approaches that were used to address model fairness and their rationale	6
Model output	15	D	Specify the output of the prediction model (e.g., probabilities, classification). Provide details and rationale for any classification and how the thresholds were identified	6

[1] D=items relevant only to the development of a prediction model; E=items relating solely to the evaluation of a prediction model; D;E=items applicable to both the development and evaluation of a prediction model

[2] Separately for all model building approaches.

[3] TRIPOD-Cluster is a checklist of reporting recommendations for studies developing or validating models that explicitly account for clustering or explore heterogeneity in model performance (eg, at different hospitals or centres). Debray et al, BMJ 2023; 380: e071018 [DOI: 10.1136/bmj-2022-071018]

Training versus evaluation	16	D;E	Identify any differences between the development and evaluation data in healthcare setting, eligibility criteria, outcome, and predictors	6
Ethical approval	17	D;E	Name the institutional research board or ethics committee that approved the study and describe the participant-informed consent or the ethics committee waiver of informed consent	4
**OPEN SCIENCE**				
Funding	18a	D;E	Give the source of funding and the role of the funders for the present study	12
Conflicts of interest	18b	D;E	Declare any conflicts of interest and financial disclosures for all authors	12
Protocol	18c	D;E	Indicate where the study protocol can be accessed or state that a protocol was not prepared	
Registration	18d	D;E	Provide registration information for the study, including register name and registration number, or state that the study was not registered	12
Data sharing	18e	D;E	Provide details of the availability of the study data	12
Code sharing	18f	D;E	Provide details of the availability of the analytical code[4]	12
**PATIENT & PUBLIC INVOLVEMENT**				
Patient & Public Involvement	19	D;E	Provide details of any patient and public involvement during the design, conduct, reporting, interpretation, or dissemination of the study or state no involvement.	12
**RESULTS**				
Participants	20a	D;E	Describe the flow of participants through the study, including the number of participants with and without the outcome and, if applicable, a summary of the follow-up time. A diagram may be helpful.	6
	20b	D;E	Report the characteristics overall and, where applicable, for each data source or setting, including the key dates, key predictors (including demographics), treatments received, sample size, number of outcome events, follow-up time, and amount of missing data. A table may be helpful. Report any differences across key demographic groups.	6
	20c	E	For model evaluation, show a comparison with the development data of the distribution of important predictors (demographics, predictors, and outcome).	6
Model development	21	D;E	Specify the number of participants and outcome events in each analysis (e.g., for model development, hyperparameter tuning, model evaluation)	7
Model specification	22	D	Provide details of the full prediction model (e.g., formula, code, object, application programming interface) to allow predictions in new individuals and to enable third-party evaluation and implementation, including any restrictions to access or re-use (e.g., freely available, proprietary)[5]	7
Model performance	23a	D;E	Report model performance estimates with confidence intervals, including for any key subgroups (e.g., sociodemographic). Consider plots to aid presentation.	7
	23b	D;E	If examined, report results of any heterogeneity in model performance across clusters. See TRIPOD Cluster for additional details[3].	8
Model updating	24	E	Report the results from any model updating, including the updated model and subsequent performance	8
**DISCUSSION**				
Interpretation	25	D;E	Give an overall interpretation of the main results, including issues of fairness in the context of the objectives and previous studies	9
Limitations	26	D;E	Discuss any limitations of the study (such as a non-representative sample, sample size, overfitting, missing data) and their effects on any biases, statistical uncertainty, and generalizability	11
Usability of the model in the context of current care	27a	D	Describe how poor quality or unavailable input data (e.g., predictor values) should be assessed and handled when implementing the prediction model	10
	27b	D	Specify whether users will be required to interact in the handling of the input data or use of the model, and what level of expertise is required of users	9
	27c	D;E	Discuss any next steps for future research, with a specific view to applicability and generalizability of the model	10

From: Collins GS, Moons KGM, Dhiman P, et al. *BMJ* 2024;385:e078378. doi:10.1136/bmj-2023-078378

[4] This relates to the analysis code, for example, any data cleaning, feature engineering, model building, evaluation.
[5] This relates to the code to implement the model to get estimates of risk for a new individual.

Just Accepted papers have undergone full peer review and have been accepted for publication. This article will undergo copyediting, layout, and proof review before it is published in its final version. Please note that during production of the final copyedited article, errors may be discovered which could affect the content.

7. Kocak B, Keles A, Akinci D'Antonoli T. Self-reporting with checklists in artificial intelligence research on medical imaging: a systematic review based on citations of CLAIM. Eur Radiol 2024;34(4):2805–2815.

8. Tejani AS, Klontzas ME, Gatti AA, et al. Updating the Checklist for Artificial Intelligence in Medical Imaging (CLAIM) for reporting AI research. Nat Mach Intell 2023;5(9):950–951.

9. Altman DG, Simera I, Hoey J, Moher D, Schulz K. EQUATOR: reporting guidelines for health research. Lancet 2008;371(9619):1149–1150.

10. Simera I, Moher D, Hirst A, Hoey J, Schulz KF, Altman DG. Transparent and accurate reporting increases reliability, utility, and impact of your research: reporting guidelines and the EQUATOR Network. BMC Med 2010;8(1):24.

11. Cohen JF, Korevaar DA, Altman DG, et al. STARD 2015 guidelines for reporting diagnostic accuracy studies: explanation and elaboration. BMJ Open 2016;6(11):e012799.

12. Gasser U. An EU landmark for AI governance. Science 2023;380(6651):1203.

13. European Commission. European Health Data Space. https://health.ec.europa.eu/ehealth-digital-health-and-care/european-health-data-space_en. Accessed March 2023.

14. Kohn MA, Senyak J. Sample size calculators. https://sample-size.net/. Accessed February 2024.

15. Reinke A, Tizabi MD, Baumgartner M, et al. Understanding metric-related pitfalls in image analysis validation. Nat Methods 2024;21(2):182–194.

16. Maier-Hein L, Reinke A, Godau P, et al. Metrics reloaded: recommendations for image analysis validation. Nat Methods 2024;21(2):195–212.

17. International Committee of Medical Journal Editors. Clinical Trials. http://www.icmje.org/recommendations/browse/publishing-and-editorial-issues/clinical-trial-registration.html. Accessed April 2024.

18. Megahed FM, Chen YJ, Megahed A, Ong Y, Altman N, Krzywinski M. The class imbalance problem. Nat Methods 2021;18(11):1270–1272.

19. Fu GH, Yi LZ, Pan J. Tuning model parameters in class-imbalanced learning with precision-recall curve. Biom J 2019;61(3):652–664.

Checklist for Artificial Intelligence in Medical Imaging (CLAIM): 2024 Update

Section/Topic	No.	Item	Yes*	No	NA
TITLE/ABSTRACT					
	1	Identification as a study of AI methodology, specifying the category of technology used (eg, deep learning)	1		
ABSTRACT					
	2	Summary of study design, methods, results, and conclusions	1		
INTRODUCTION					
	3	Scientific and/or clinical background, including the intended use and role of the AI approach	2		
	4	Study aims, objectives, and hypotheses	3		

This copy is for personal use only.

Just Accepted papers have undergone full peer review and have been accepted for publication. This article will undergo copyediting, layout, and proof review before it is published in its final version. Please note that during production of the final copyedited article, errors may be discovered which could affect the content.

METHODS					
Study Design	5	Prospective or retrospective study	3		
	6	Study goal	3		
Data	7	Data sources	3		
	8	Inclusion and exclusion criteria	4		
	9	Data preprocessing	4		
	10	Selection of data subsets	4		
	11	De-identification methods	5		
	12	How missing data were handled	5		
	13	Image acquisition protocol	4		
Reference Standard	14	Definition of method (s) used to obtain reference standard	4		
	15	Rationale for choosing the reference standard	4		
	16	Source of reference standard annotations Statistical measures of significance and uncertainty	4		
	17	Annotation of test set	4		
	18	Measures of inter-and intrarater variability of features described by the annotators	4		
Data Partitions	19	How data were assigned to partitions	4		
	20	Level at which partitions are disjoint	4		
Testing Data	21	Intended sample size	4		
Section/Topic	No.	Item	Yes*	No	NA
Model	22	Detailed description of model	5		
	23	Software libraries, frameworks, and packages	5		
	24	Initialization of model parameters	5		
Training	25	Details of training approach	6		
	26	Method of selecting the final model	6		
	27	Ensembling techniques	6		
Evaluation	28	Metrics of model performance	6		
	29	Statistical measures of significance and uncertainty	6		
	30	Robustness or sensitivity analysis	6		
	31	Methods for explainability or interpretability	6		
	32	Evaluation on internal data	6		
	33	Testing on external data	6		
	34	Clinical trial registration	6		
RESULTS					
Data	35	Numbers of patients or examinations included and excluded	6		
	36	Demographic and clinical characteristics of cases in each partition	6		
Model performance	37	Performance metrics and measures of statistical uncertainty	7		
	38	Estimates of diagnostic performance and their precision	8		
	39	Failure analysis of incorrectly classified cases	8		
DISCUSSION					
	40	Study limitations	11		
	41	Implications for practice, including intended use and/or clinical role	9		
OTHER INFORMATION					
	42	Provide a reference to the full study protocol or to additional technical details	12		
	43	Statement about the availability of software, trained model, and/or data	12		
	44	Sources of funding and other support; role of funders	12		

* Indicate page and/or line number for each checklist item that is present.

This copy is for personal use only.
